# Accuracy of calculating mechanical power of ventilation by one commonly used equation

**DOI:** 10.1007/s10877-022-00823-3

**Published:** 2022-04-15

**Authors:** Shin-Hwar Wu, Chew-Teng Kor, I.-Chieh Mao, Chun-Ching Chiu, Kai-Huang Lin, Cheng-Deng Kuo

**Affiliations:** 1 Division of Critical Care Internal Medicine, Department of Emergency Medicine and Critical Care, Changhua Christian Hospital, Changhua, Taiwan; 2 Medical College, National Chung Hsing University, Taichung, Taiwan; 3 Big Data Center, Changhua Christian Hospital, Changhua, Taiwan; 4 Graduate Institute of Statistics and Information Science, National Changhua University of Education, Changhua, Taiwan; 5 Department of Medical Research, Taipei Veterans General Hospital, No. 201, Sec. 2, Shihpai Rd, Beitou District, Taipei, 112 Taiwan; 6 Division of Chest Medicine, Department of Internal Medicine, Changhua Christian Hospital, Changhua, Taiwan; 7 Tanyu Research Laboratory, Taipei, Taiwan

**Keywords:** Acute respiratory distress syndrome, Mechanical power, Mechanical ventilation, Positive end-expiratory pressure, Respiratory failure, Ventilator-induced lung injury, Work of breathing

## Abstract

Gattinoni’s equation, ${\text{MP}}_{{{\text{rs}}}} \, = \,0.098\, \times \,{\text{RR}}\, \times \,\left[ {{\text{V}}_{T} ^{2} \left( {\frac{1}{2}{\text{E}}_{{{\text{rs}}}} \, + \,{\text{RR}}\, \times \,\frac{{1\, + \,I:E}}{{60\, \times \,I:E}}\, \times \,{\text{R}}_{{{\text{aw}}}} } \right)\, + \,{\text{V}}_{{\text{T}}} \, \times \,{\text{PEEP}}} \right]$, now commonly used to calculate the mechanical power (MP) of ventilation. However, it calculates only inspiratory MP. In addition, the inclusion of PEEP in Gattinoni’s equation raises debate because PEEP does not produce net displacement or contribute to MP. Measuring the area within the pressure–volume loop accurately reflects the MP received in a whole ventilation cycle and the MP thus obtained is not influenced by PEEP. The MP of 25 invasively ventilated patients were calculated by Gattinoni’s equation and measured by integration of the areas within the pressure–volume loops of the ventilation cycles. The MP obtained from both methods were compared. The effects of PEEPs on MP were also evaluated. We found that the MP obtained from both methods were correlated by R^2^ = 0.75 and 0.66 at PEEP 5 and 10 cmH_2_O, respectively. The biases of the two methods were 3.13 (2.03 to 4.23) J/min (P < 0.0001) and − 1.23 (− 2.22 to − 0.24) J/min (P = 0.02) at PEEP 5 and 10 cmH_2_O, respectively. These P values suggested that both methods were significantly incongruent. When the tidal volume used was 6 ml/Kg, the MP by Gattinoni’s equation at PEEP 5 and 10 cmH_2_O were significantly different (4.51 vs 7.21 J/min, P < 0.001), but the MP by PV loop area was not influenced by PEEPs (6.46 vs 6.47 J/min, P = 0.331). Similar results were observed across all tidal volumes. We conclude that the Gattinoni’s equation is not accurate in calculating the MP of a whole ventilatory cycle and is significantly influenced by PEEP, which theoretically does not contribute to MP.

**Supplementary Information:**

The online version contains supplementary material available at 10.1007/s10877-022-00823-3.

## Introduction

In 2016, Gattinoni et al. proposed a hypothesis that mechanical power (MP) delivered to the mechanically ventilated patients contributes to their ventilator-induced lung injury (VILI). Since the MP is hard to measure directly in clinical practice, they invented a formula to estimate the MP by algebraic calculation. Their equation incorporates several routinely monitored ventilator parameters and is written as:

$${\text{MP}}_{{{\text{rs}}}} \, = \,0.098\, \times \,{\text{RR}} \times \left[ {{\text{V}}_{{\text{T}}} ^{2} \left( {\frac{1}{2}{\text{E}}_{{{\text{rs}}}} \, + \,{\text{RR}}\, \times \,\frac{{1\, + \,I:E}}{{60\, \times \,I:E}}\, \times \,{\text{R}}_{{{\text{aw}}}} } \right)\, + \,{\text{V}}_{{\text{T}}} \, \times \,{\text{PEEP}}} \right],$$

where ${\text{MP}}_{\text{rs}}$ is the MP received by the respiratory system, 0.098 is the conversion factor from $\text{L*cmH}_{2}\text{O}$ to Joule, RR is respiratory rate, V_T_ is the tidal volume, E_rs_ is the elastance of the respiratory system, I: E is the inspiration to expiration ratio, R_aw_ represents airway resistance and PEEP is the value of positive end-expiratory pressure. The equation can also be simplified to read.

$${\text{MP}}_{{{\text{rs}}}} \, = \,0.098\, \times \,{\text{RR}}\, \times \,{\text{V}}_{{\text{T}}} \, \times \,\left[ {{\text{P}}_{{{\text{peak}}}} \, - \,\frac{1}{2}\left( {{\text{P}}_{{{\text{plat}}}} \, - \,{\text{PEEP}}} \right)} \right],$$

where P_peak_ is the peak inspiratory airway pressure and P_plat_ is the plateau pressure. Please refer to their original article for details of derivation of these equations [1].

Since its publication, this calculation equation has gained wide acceptance. It has been adopted as a reference standard for comparison with new MP estimation methods [2, 3]. It was also commonly used in clinical studies to examine the correlation between MP and outcomes of various kinds of patients [4–6].

**Fig. 1:**
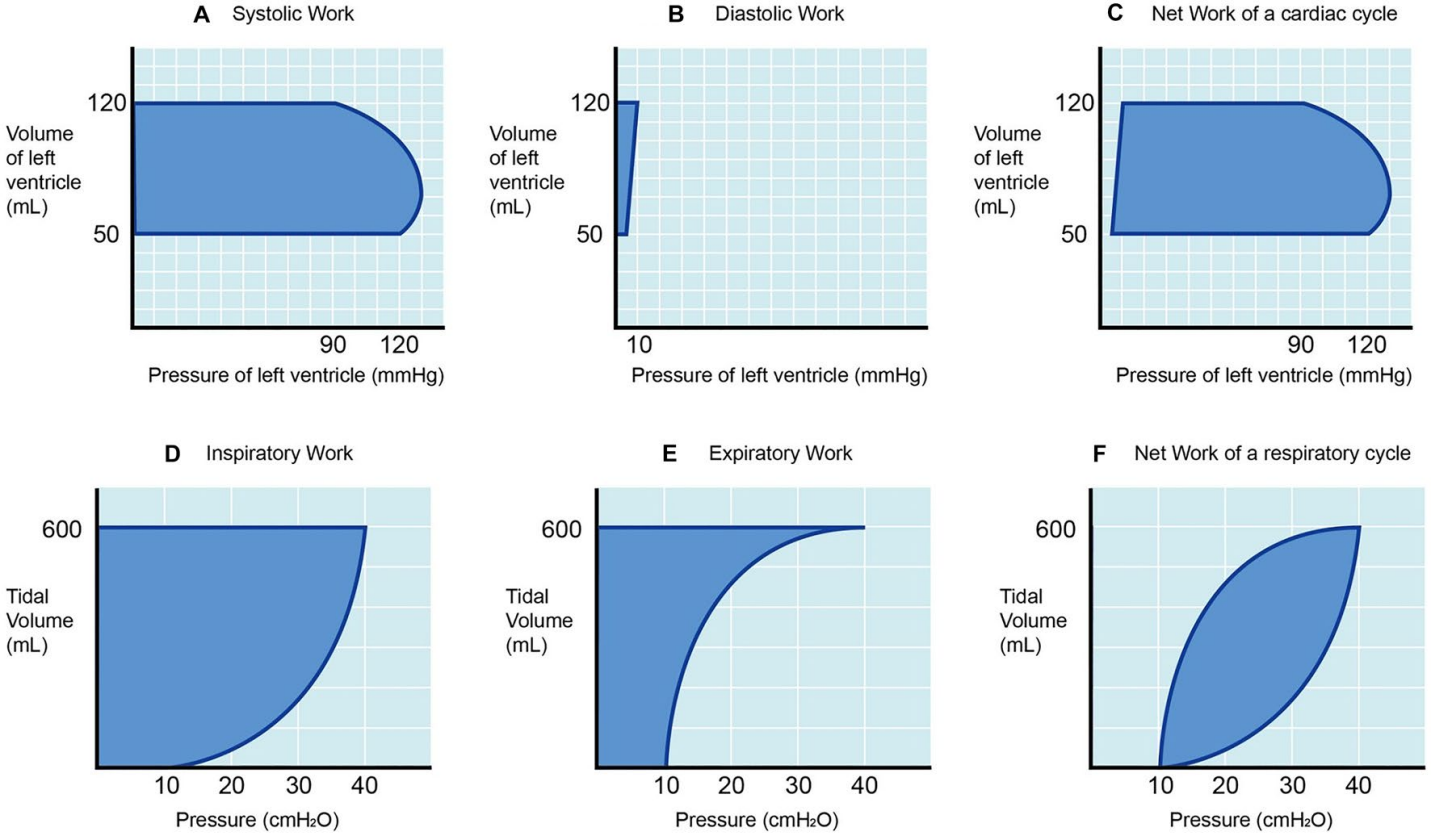
The work done by a physiologic pathway with hysteresis is the area enclosed by a PV loop. The work during left ventricular systole is shown in (**A**), and the work during left ventricular diastole is shown in (**B**). Net work of a cardiac cycle is the work of systole subtracting that of diastole (**C**). By the same principle, the work done by inspiration (**D**) subtracting that of expiration (**E**) results in a net work of a respiratory cycle (**F**). Both net works of a cardiac and respiratory cycle are the hysteresis areas of PV loops

Nevertheless, Gattinoni’s equation calculates only the work of inspiration. It was verified by comparing with the measured area between inspiratory pressure curve and volume axis, which represents the inspiratory work only (Fig. 1D). Gattinoni admitted that expiration ‘very possibly’ also contributes to MP [7, 8]. Actually, expiration is an integral part of a respiratory cycle and exerts its own mechanical work in a direction opposite to that of inspiration. So, the network of a tidal ventilation is obtained by subtracting the expiratory from the inspiratory works (Fig. 1E and F). Mechanical work of a complete tidal ventilation cycle, including both inspiratory and expiratory parts, is graphically represented by the hysteresis area surrounded by a pressure–volume (PV) loop [9, 10]. For any displacement with hysteresis, the work done is measured by the area enclosed in the loop of the movement path. This principle also holds true in the realms of thermodynamics [11] and cardiac physiology [12]. Theoretically, this measurement of mechanical work (or MP) is more accurate than those considering only one limb of hysteresis.

Considering only inspiratory limb also raises debate about including PEEP value in the calculation of work [13]. The work is defined by either force × distance, or pressure × volume. Since PEEP does not by itself produce displacement or volume change during tidal ventilation, its contribution to work is theoretically zero. If both inspiration and expiration are considered and work of ventilation is measured by the hysteresis area surrounded by PV loop, PEEP plays no role at all.

In this study, we compared MPs calculated from Gattinoni’s equation with those obtained from measuring the hysteresis area surrounded by the PV loop to evaluate the accuracy of this equation.

## Methods

### Study design

Invasively ventilated patients admitted to the intensive care units of Changhua Christian Hospital (Changhua, Taiwan) from Aug. 2019 to Apr. 2021 were prospectively enrolled. We excluded patients under 20 or over 90 years of age, with over 60% of the inspired fraction of O_2_, with Acute Physiology and Chronic Health Evaluation score over 30, with a plateau pressure over 30 cmH_2_O, or with unstable hemodynamics. The study was approved by the institutional review board of Changhua Christian Hospital (Approval No. 181262). Informed consents were obtained from surrogates of all participants.

### Raw data acquisition

All patients were measured by an Evita 4 ventilator (Dräger Medical, Lübeck, Germany), which was connected to a laptop with Ventview (Dräger Medical, Lübeck, Germany) software for data collection. Appropriate sedation and muscle relaxation were given to patients with spontaneous respiration. Upon measurement, ventilators were set at volume-controlled mode with a constant flow which was adjusted to avoid harmfully high airway pressure. Inspiratory time was lengthened to generate an inspiratory hold long enough for measuring plateau pressure. Various combinations of V_T_ (6, 8, and 10 ml per kg of body weight) and PEEPs (5 and 10 cmH_2_O) were set during measurement. Raw data of airway pressure, flow and volume during the measurement were downloaded to the laptop via Medibus protocol with a sampling rate of 67 Hz for subsequent offline analysis.

### Derivation and calculation of ventilation-related parameters

The P_plat_ was defined by the last pressure value at the inspiratory plateau phase with zero airflow. The PEEP value was defined by the average of all pressure data of expiratory phase starting from flow drops to − 1 L/min. The E_rs_, R_aw_, RR, V_T_, I: E and the area inside the PV loop were calculated from the downloaded raw data breath by breath using an executable program written with MATLAB 7.2 (The MathWorks, Natick, MA, USA). The work of ventilation was assessed by both Gattinoni’s equation [1] and PV loop area. The work expressed in ${\text{L}}\,{\text{cmH}}_{2} {\text{O}}$ was converted to Joules by a factor of 0.098. The MP was obtained by multiplying the work per breath by the RR.

### Statistical analysis

Data were expressed as number (percentage) for categorical data or median and interquartile range for continuous data. A simple regression model was performed to evaluate the correlation between MP values by Gattinoni’s equation and PV loop area. Bland–Altman analysis was used to evaluate the agreement between the two MP evaluation methods. Wilcoxon signed-rank test was used to compare two repeatedly measured works at PEEP levels of 5 and 10 cmH_2_O. The SAS 9.4 software (SAS Institute Inc., Cary, NC, USA) was used for statistical analysis. A two-tailed *p* value less than 0.05 was considered statistically significant.

## Results

### MP by Gattinoni’s equation and PV loop area

**Fig. 2:**
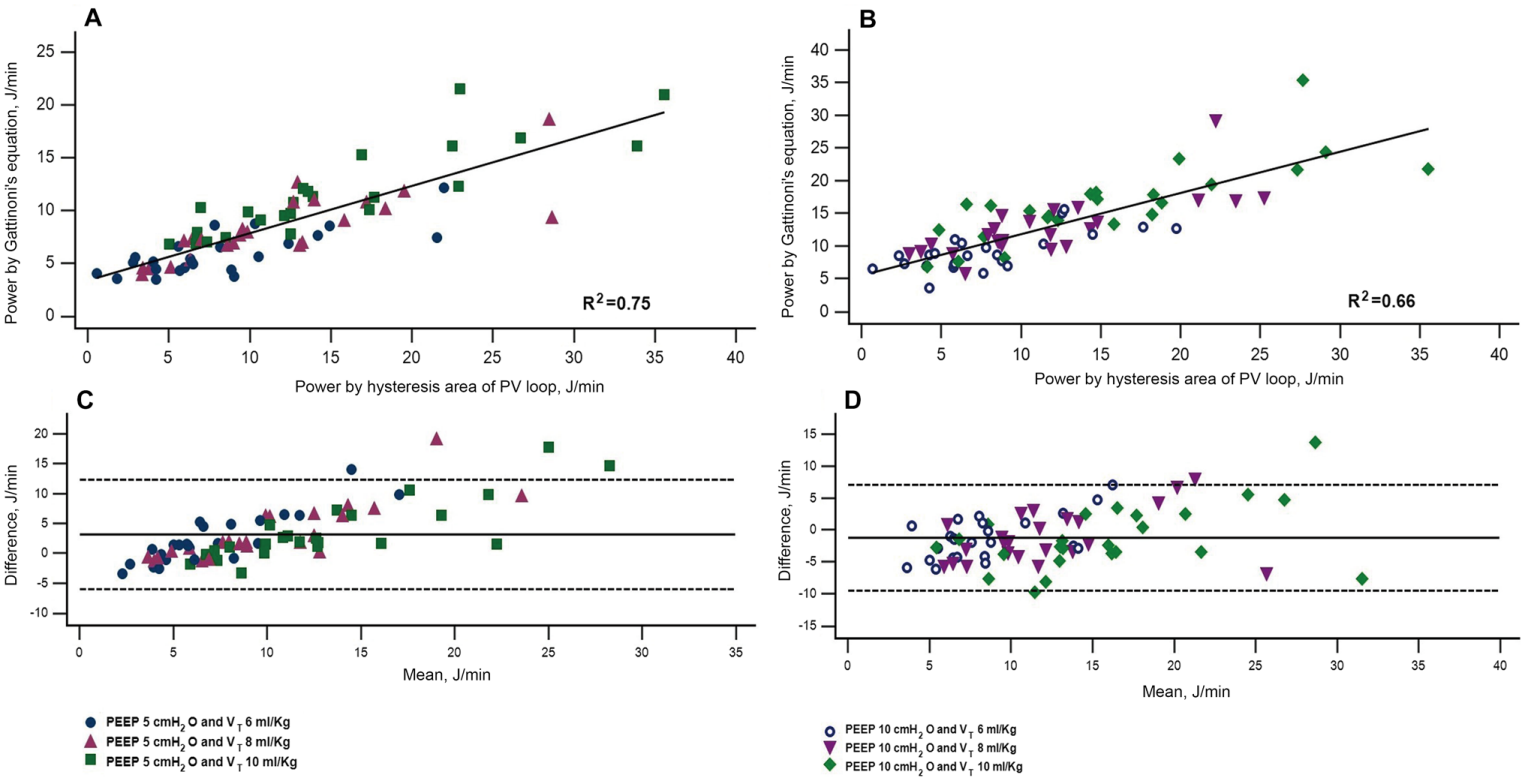
Simple regression models expressing the correlations between MPs by both methods at PEEP 5 (**A**) and 10 cmH_2_O (**B**). Bland–Altman plots depicting differences between the two methods of evaluating MP at PEEP 5 (**C**) and 10 cmH_2_O (**D**). The differences were more prominent when the mean MP was larger than 15 J/min

**Fig. 3:**
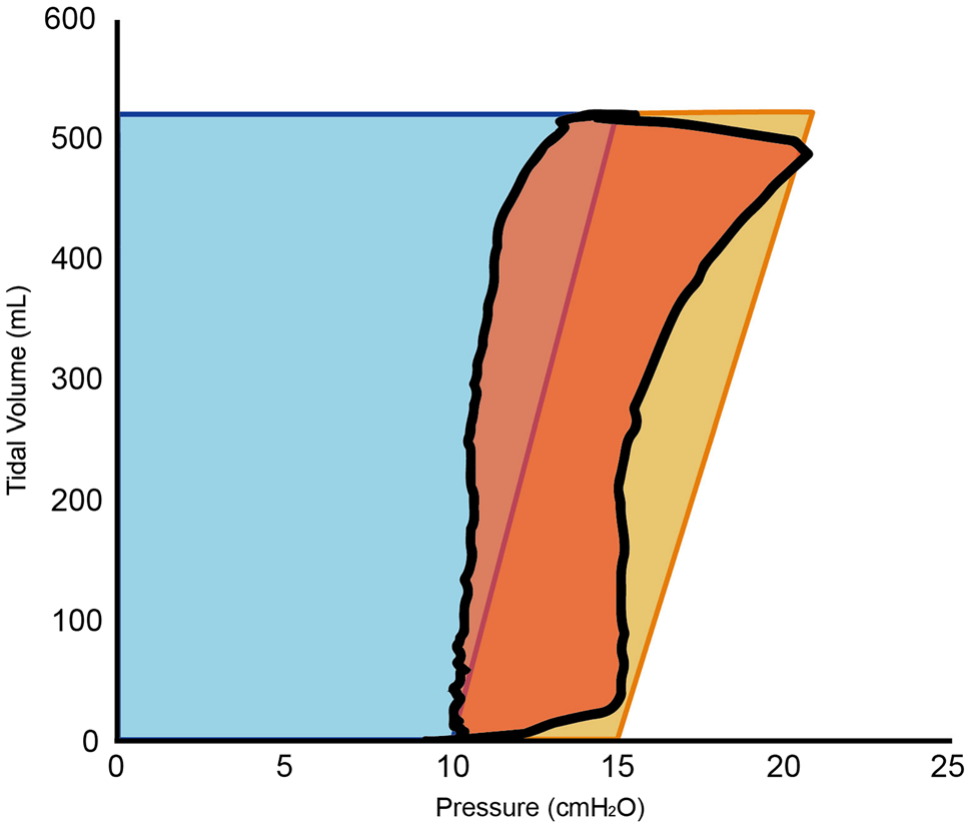
One patient’s PV loop (orange area) at V_T_ 10 ml/Kg and PEEP 10 cmH_2_O was plotted against the calculated area (elastic work in blue plus resistive work in yellow) by the Gattinoni’s equation. A prominent incongruence of both areas can be easily observed. The work of ventilation by hysteresis area of PV loop was 0.27 J, while the work by Gattinoni’s equation was 0.70 J

A total of 25 patients were enrolled and their baseline clinical characteristics were listed in Table 1. When the PEEP was 5 cmH_2_O, the MPs by both methods were correlated by a regression formula: MP by Gattinoni’s equation = 3.37 + 0.45 × MP by PV loop area, R^2^ = 0.75, P < 0.001 (Fig. 2A). When the PEEP was 10 cmH_2_O, the regression formula was: MP by Gattinoni’s equation = 5.51 + 0.63 × MP by PV loop area, R^2^ = 0.66, P < 0.001 (Fig. 2B). The Bland–Altman plot corresponding to PEEP 5 cmH_2_O was presented in Fig. 2C. The mean of difference was 3.13 J/min, and the 95% confidence interval was 2.03 to 4.23 J/min (lower limit = − 6.05 J/min, upper limit = 12.30 J/min). The P value for the null hypothesis (H_0_: mean of difference = 0) was less than 0.0001, which means that the results of both methods were significantly different. The Bland–Altman plot of PEEP 10 cmH_2_O was presented in Fig. 2D. The mean of difference was − 1.23 J/min, and the 95% confidence interval was − 2.22 to − 0.24 J/min (lower limit = − 9.47 J/min, upper limit = 7.01 J/min). The P value for the null hypothesis was 0.02, suggesting that the results of both methods were significantly different. The largest differences tended to be found when the mean MP was greater than 15 J/min. One of our patient’s PV loop at V_T_ 10 ml/Kg and PEEP 10 cmH_2_O was plotted against the calculated area by Gattinoni’s equation (Fig. 3). A prominent incongruence of both areas can be easily observed. When the whole study population was divided into those with and without acute respiratory distress syndrome (ARDS), the results were similar except for ARDS patients under PEEP 10 cmH_2_O where no difference could be found probably because the case number of ARDS was too small (only 7). These data could be found in our supplemental Figs. s1 and s2.

**Table 1:** Baseline characteristics of enrolled patients (n = 25)

Variables	Number (%) or Median (IQR)
Age (year)	63 (52–75)
Male (n, %)	17 (68%)
Height (cm)	160 (156–169)
Body Mass Index (Kg/m^2^)	24.68 (22.73–27.78)
ARDS (Y/N)	7/18
Respiratory rate (min^−1^)	18 (14–20)
Ventilator mode PCV: VCV	22:3
Monitored V_T_ (ml)	542 (500–603)
Driving pressure (cmH_2_O)	20 (18–22)
PEEP (cmH_2_O)	6 (5–8)
Static compliance (ml/cmH_2_O)	27.1 (23.81–31.25)
P_a_O_2_/F_I_O_2_ (mmHg)	271 (136–361)
A-a DO_2_ (mmHg)	185 (96–315)
PaCO2 (mmHg)	32 (25–39)

*A-a DO2* alveolar-arterial oxygen difference, *PCV* pressure-controlled ventilation, *VCV* volume-controlled ventilation

### Assessing effects of PEEP on MP by both methods

Because PEEP does not by itself produce any air displacement during tidal ventilation, its contribution to MP is theoretically zero. The MPs, by integration of the areas inside the PV loop, were not significantly different at PEEP 5 and PEEP 10 cmH_2_O. On the other hand, the MPs calculated by Gattinoni’s equation, at PEEP 5 cmH_2_O were significantly smaller than those at PEEP 10 cmH_2_O (Table 2). The MPs by Gattinoni’s equation of ARDS patients did not differ significantly at PEEP 5 and 10 cmH_2_O. This may be due to small case numbers in this group.

**Table 2:** Assessing effects of PEEP on mechanical power by Gattinoni’s equation and area of PV loop

	Power by Gattinoni Equation (J/min)			Power by hysteresis area of PV loop (J/min)		
	PEEP 5 cmH_2_O	PEEP 10 cmH_2_O	P	PEEP 5 cmH_2_O	PEEP 10 cmH_2_O	P
Non-ARDS (n = 18)						
V_T_ 6 ml/kg	5.07 (4.34, 7.56)	8.50 (6.94, 10.38)	< 0.001	6.51 (4.22, 9.69)	6.29 (4.25, 10.27)	0.381
V_T_ 8 ml/kg	7.15 (6.04, 9.82)	10.62 (9.63, 15.06)	0.001	9.56 (6.13, 13.16)	8.82 (7.24, 13.82)	0.868
V_T_ 10 ml/kg	10.08 (7.69, 13.77)	14.81 (12.9, 19.98)	< 0.001	13.29 (7.94, 20.28)	12.30 (8.56, 19.38)	0.435
ARDS (n = 7)						
V_T_ 6 ml/kg	2.63 (1.27, 3.44)	2.80 (0.6, 4.72)	0.735	6.41 (2.94, 12.38)	8.50 (4.59, 12.69)	0.612
V_T_ 8 ml/kg	4.48 (3.26, 6.76)	4.44 (2.79, 6.44)	0.612	13.99 (6.44, 17.21)	11.83 (8.32, 14.34)	0.091
V_T_ 10 ml/kg	6.59 (4.58, 11.63)	7.80 (5.94, 8.93)	1	12.67 (10.68, 22.48)	14.80 (6.58, 21.97)	0.735
All (n = 25)						
V_T_ 6 ml/kg	4.51 (3.46, 7.16)	7.21 (3.85, 9.49)	< 0.001	6.46 (4.21, 10.48)	6.47 (4.33, 10.84)	0.331
V_T_ 8 ml/kg	6.84 (4.62, 9.12)	9.97 (6.09, 13.68)	0.001	9.71 (6.35, 15.38)	9.71 (8.05, 14.15)	0.361
V_T_ 10 ml/kg	9.76 (6.82, 12.25)	13.68 (8.28, 16.49)	0.001	12.98 (8.86, 21.28)	14.52 (8.34, 19.65)	0.607

Wilcoxon Signed Rank Test is a non-parametric statistical hypothesis test that compares two repeated measurements of mechanical power values. It is used to assess whether the average mechanical power rank is different between PEEP 5 and PEEP 10 cmH_2_O

## Discussion

In this study, we provided evidence to challenge the accuracy and validity of Gattinoni’s equation. In his original publication, the MPs by Gattinoni’s equation were in very good correlation with the measured ones by R^2^ of 0.96 to 0.99 and the mean biases between the two methods were minimal (around ± 0.5 J/min) [1]. In contrast, our data revealed a less than perfect agreement between MPs by Gattinoni’s equation and measured ones. This discrepancy lies in the difference in MP measurement. Gattinoni’s measured areas between inspiratory pressure curve and ordinate of volume, but we measured areas surrounded by the PV loop. Gattinoni’s measurement was restricted to the inspiratory phase, while ours took both inspiration and expiration into account and was a more reliable value of the MP received during the tidal ventilatory cycle.

There is growing awareness of the important role played by expiration during a ventilatory cycle. The MP accumulated at end-inspiration is eliminated by exhaling into the atmosphere or dissipating into lung tissue during the expiratory phase [7]. Therefore, expiration surely contributes to MP and the consequential VILI. By manipulating the expiratory flow of mechanical ventilation, we can achieve more lung recruitment [14], more homogeneous lung aeration [15], better gas exchange [16], and less VILI [17]. So, neglecting expiration makes Gattinoni’s equation prone to inaccuracy in assessing the MP that a ventilated patient received.

We think the most crucial origin of inaccuracy stems from including PEEP in the calculating equation of Gattinoni’s et al. Our data demonstrated that the PEEP value did not influence MP measured by PV loop method, but it falsely increased the MP calculated by using Gattinoni’s equation (Table 2). As we mentioned in the introduction, incorporating a static pressure without net displacement, like PEEP, into the calculation of MP is contradictory to the basic law of physics. Moreover, Gattinoni’s equation suggested that a high PEEP can increase both the MP and the chance of subsequent VILI. However, this assumption could find support from neither animal studies nor clinical trials. High PEEPs failed to produce evidence of VILI in the lungs of the experimental animals [18, 19]. According to a metanalysis of 8 randomized trials on ARDS patients, high PEEPs did not lead to worse clinical outcomes and can even reduce some patients’ mortality [20].

Gattinoni’s original hypothesis that MP induces VILI and subsequent poor clinical outcomes has never been unequivocally proved [8]. Studies on the clinical implications of MP were currently all observational. Some studies showed a good correlation between MP and mortality [21, 22], whereas some did not [23, 24]. Still, some studies barely established a correlation by modifying the definition of MP calculated [4, 25].

Proposing a revised version of Gattinoni’s equation is beyond the scope of this article. It cannot be done by simply subtracting PEEP from the equation. We have tried to calculate the MP of our patients by using Gattinoni’s equation without PEEP term, but the results are still unsatisfactory (Please refer to our supplement Fig. s3).

There are two limitations to our study that worth mentioning. First, our ARDS patient number was too small to draw a solid conclusion regarding this subgroup. The insignificant results from analysis within this group are all subject to type II error. Second, to avoid harmful VILI, we did not apply a V_T_ of more than 10 ml/Kg or a PEEP over 10 cmH_2_O during MP measurement. Extrapolating our results beyond these V_T_ or PEEP limitations is subject to imprecision.

In conclusion, Gattinoni’s equation is not accurate in the calculation of the MP during a whole ventilatory cycle and is significantly influenced by PEEP, which theoretically does not contribute to MP.

## Supplementary Information

Below is the link to the electronic supplementary material.

Supplementary file1 (DOCX 164 kb)

Supplementary file2 (DOCX 151 kb)

Supplementary file3 (DOCX 204 kb)

## Abbreviations

ARDS: Acute respiratory distress syndrome
E_rs_: Elastance of respiratory system
I:E: Inspiratory to expiratory ratio
MP: Mechanical power
PEEP: Positive end-expiratory pressure
P_peak_: Peak airway pressure
P_plat_: Plateau pressure
Raw: Airway resistance
PV: Pressure–volume
RR: Respiratory rate
VILI: Ventilator-induced lung injury
V_T_: Tidal volume
