# Supplementary material for: Accuracy of calculating mechanical power of ventilation by one commonly used equation
Source: J Clin Monit Comput. 2022 Apr 15;36(6):1753–9. doi: 10.1007/s10877-022-00823-3 (PMC9637605; doi:10.1007/s10877-022-00823-3)
Supplement: Supplementary file 1 — Supplementary file1 (DOCX 164 kb) [file 10877_2022_823_MOESM1_ESM.docx]

**
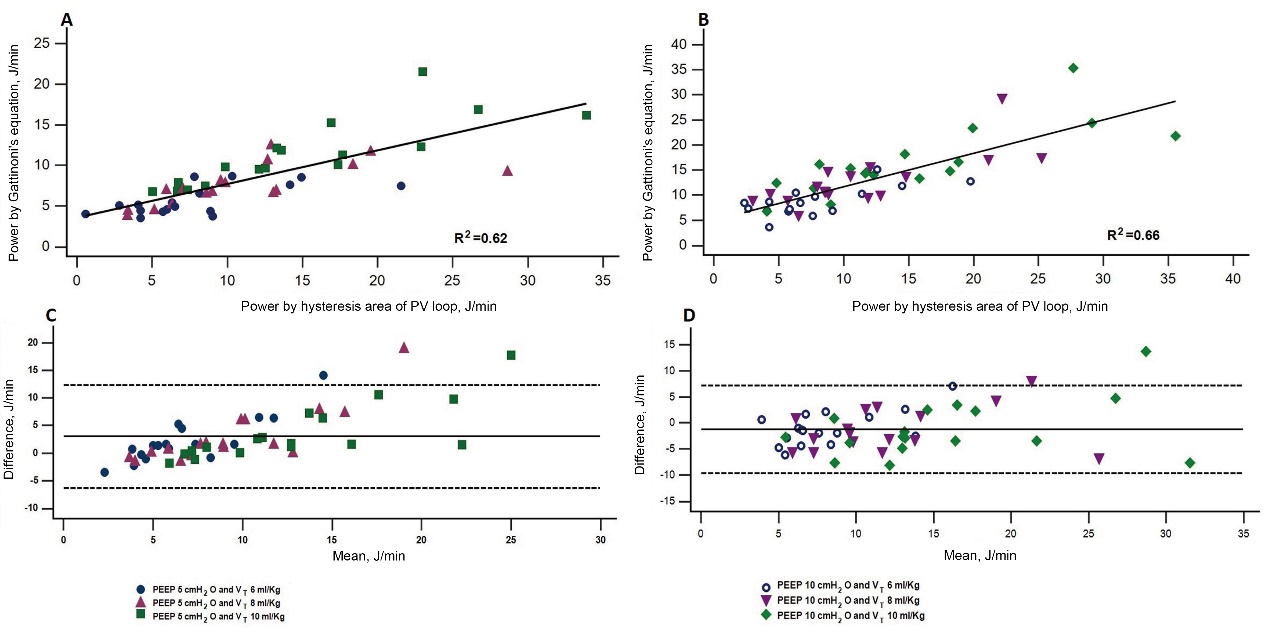
**

**Fig s1.** Simple regression models expressing correlations between computed and calculated MPs for non-ARDS patients (n = 18). The regression equation was: MP by Gattinoni equation = 3.58 + 0.41 x MP by PV loop for PEEP 5 cmH_2_O (A). The equation was: MP by Gattinoni equation = 5.02 + 0.67 x MP by PV loop for PEEP 10 cmH_2_O (B). (C) The corresponding Bland-Altman plot at PEEP 5 cmH_2_O. Mean of difference was 3.03 J/min. 95% of confidence interval was 1.69 to 4.37 J/min (lower limit = -6.30 J/min, upper limit = 12.37 J/min). P value for null hypothesis was less than 0.0001. (D) The corresponding Bland-Altman plot at PEEP 10 cmH_2_O. Mean of difference was -1.21 J/min. 95% of confidence interval was -2.42 to -0.01 J/min (lower limit = -9.62 J/min, upper limit = 7.19 J/min). P value for null hypothesis was 0.048.
