# Supplementary material for: Accuracy of calculating mechanical power of ventilation by one commonly used equation
Source: J Clin Monit Comput. 2022 Apr 15;36(6):1753–9. doi: 10.1007/s10877-022-00823-3 (PMC9637605; doi:10.1007/s10877-022-00823-3)
Supplement: Supplementary file 2 — Supplementary file2 (DOCX 151 kb) [file 10877_2022_823_MOESM2_ESM.docx]

**
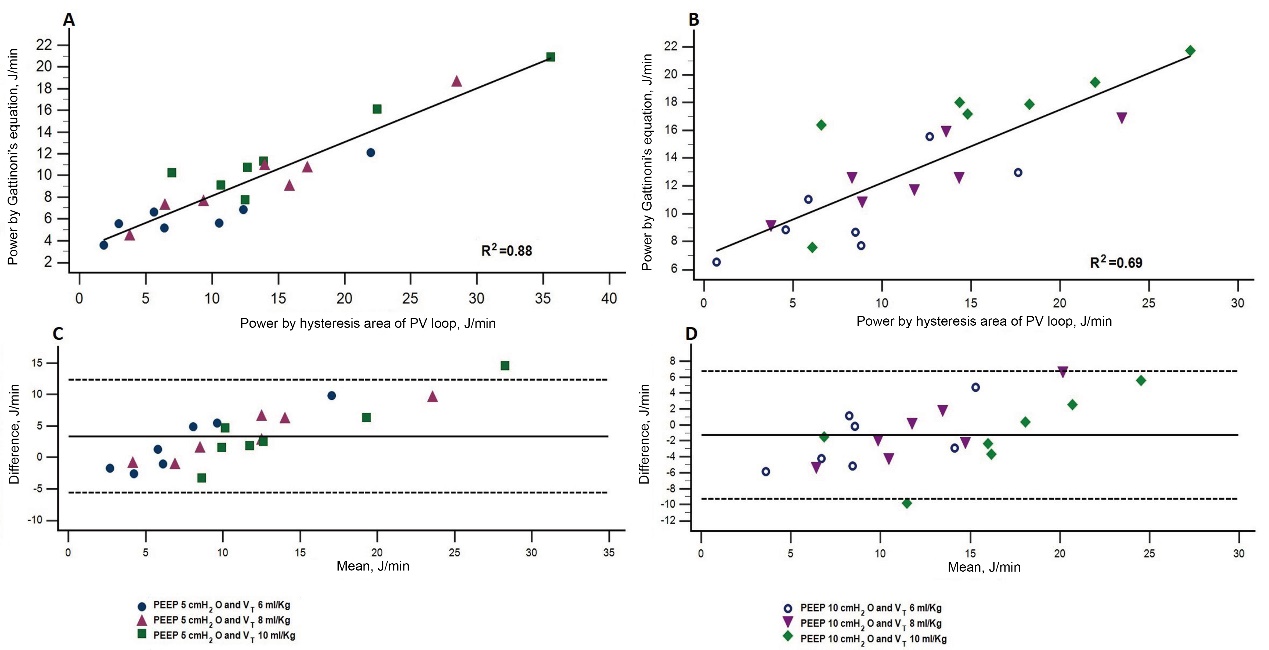
**

**Fig s2.** Simple regression models expressing correlations between computed and calculated MPs for ARDS patients (n =7). The regression equation was: MP by Gattinoni equation = 3.16 + 0.50 x MP by PV loop for PEEP 5 cmH_2_O (A). The equation was: MP by Gattinoni equation = 6.96 + 0.53 x MP by PV loop for PEEP 10 cmH_2_O (B). (C) The corresponding Bland-Altman plot at PEEP 5 cmH_2_O. Mean of difference was 3.35 J/min. 95% of confidence interval was 1.26 to 5.44 J/min (lower limit = -5.65 J/min, upper limit = 12.36 J/min). P value for null hypothesis was 0.003. (D) The corresponding Bland-Altman plot at PEEP 10 cmH_2_O. Mean of difference was -1.27 J/min. 95% of confidence interval was -3.13 to 0.59 J/min (lower limit = -9.29 J/min, upper limit = 6.74 J/min). P value for null hypothesis was 0.17.
