# Supplementary material for: Accuracy of calculating mechanical power of ventilation by one commonly used equation
Source: J Clin Monit Comput. 2022 Apr 15;36(6):1753–9. doi: 10.1007/s10877-022-00823-3 (PMC9637605; doi:10.1007/s10877-022-00823-3)
Supplement: Supplementary file 3 — Supplementary file3 (DOCX 204 kb) [file 10877_2022_823_MOESM3_ESM.docx]

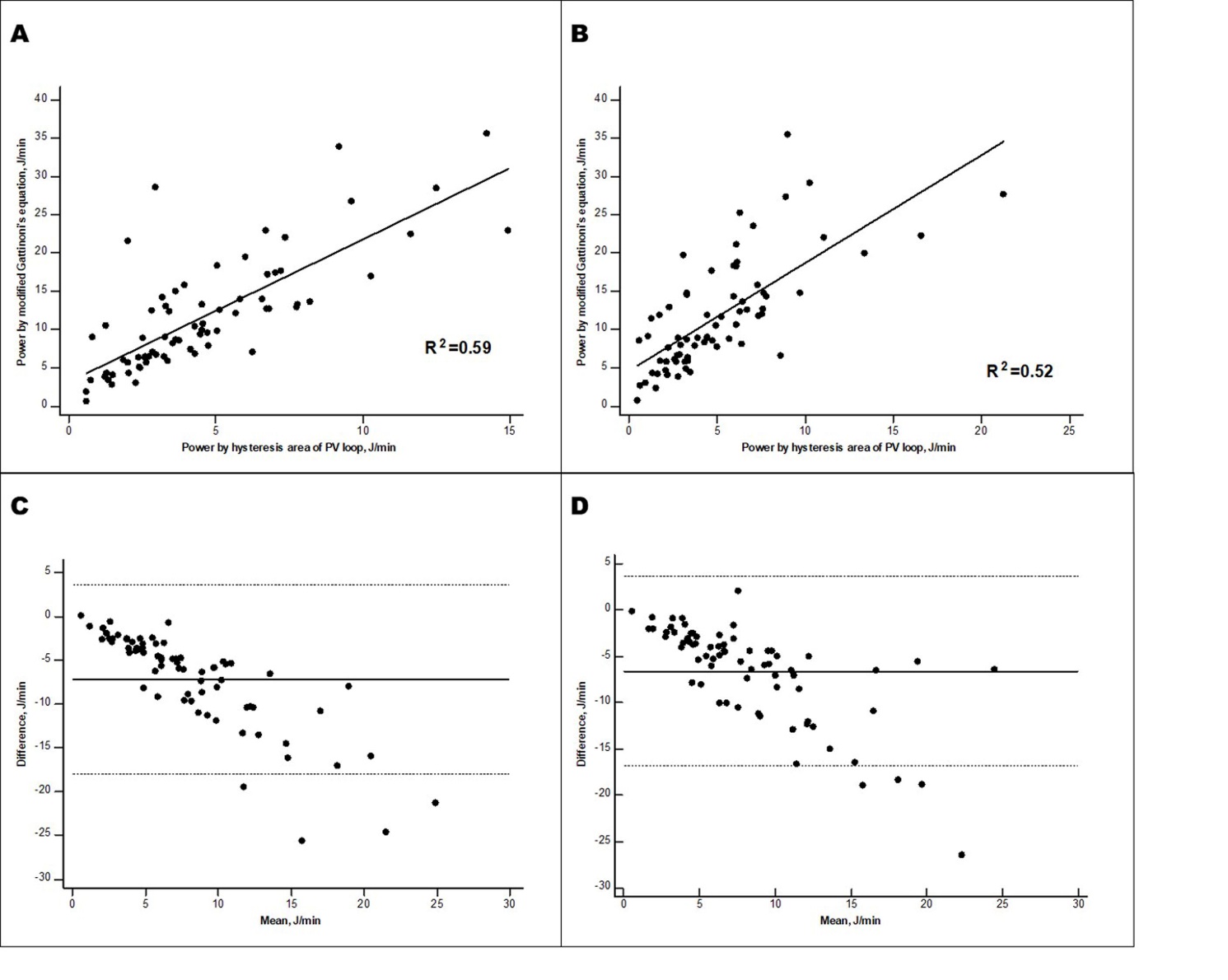


**Fig s3.** Simple regression models expressing correlations between MPs by modified Gattinoni’s equation and those by hysteresis area of PV loops. Modified Gattinoni’s equation was obtained by subtracting V_T_ x PEEP from the original one and read as ${MP}_{rs}=0.098\cdot RR\cdot\left[ {V_{T}}^{2}\left( \frac{1}{2}E_{rs}+RR\cdot\frac{1+I:E}{60\cdot I:E}{\cdot R}_{aw} \right) \right]$. The regression equation was: MP by modified Gattinoni’s equation = 3.18 + 1.86 x MP by PV loop for PEEP 5 cmH_2_O and V_T_ 6, 8, 10 ml/Kg (A). The equation was: MP by modified Gattinoni’s equation = 4.51 + 1.42 x MP by PV loop for PEEP 10 cmH_2_O and V_T_ 6, 8, 10 ml/Kg (B). (C) The corresponding Bland-Altman plot at PEEP 5 cmH_2_O. Mean of difference (PV loop – modified Gattinoni’s equation) was -7.16 J/min. (lower limit = -17.95 J/min, upper limit = 3.63 J/min). (D) The corresponding Bland-Altman plot at PEEP 10 cmH_2_O. Mean of difference was -6.61 J/min. (lower limit = -16.85 J/min, upper limit = 3.64 J/min).
